# Gene expression in blood from an individual with β‐thalassemia: An RNA sequence analysis

**DOI:** 10.1002/mgg3.740

**Published:** 2019-05-27

**Authors:** Forough Taghavifar, Mohammad Hamid, Gholamreza Shariati

**Affiliations:** ^1^ Department of Biology California State University Northridge California USA; ^2^ Department of Molecular Medicine, Biotechnology Research Center Pasteur Institute of Iran Tehran Iran; ^3^ Narges Medical Genetics & PND Laboratory Ahvaz Iran; ^4^ Department of Medical Genetics, Faculty of Medicine Ahvaz Jundishapur University of Medical Sciences Ahvaz Golestan Iran

**Keywords:** differential gene expression, hydrops fetalis, RNA‐seq, sickle cell disease, β‐thalassemia

## Abstract

**Background:**

Transcriptome profiling in individuals affected with β‐thalassemia, especially in individuals who carry novel mutations in the HBB, may improve our understanding of the heterogeneity and molecular mechanisms of the disease.

**Methods:**

Members of a family with a daughter affected with thalassemia intermedia, although her mother was not clinically affected, were examined. We also characterized genome‐wide gene expression in the family using real‐time quantitative polymerase chain reaction and high‐throughput RNA‐sequencing mRNA expression profiling of blood.

**Results:**

We described the downregulation of the β‐globin gene in β‐thalassemia by RNA‐sequencing analysis using a sample from an affected individual and her mother, who have a novel mutation in the HBB that creates a cryptic donor splice site. The daughter has a typical β‐thalassemia allele as well, and an unexpectedly severe phenotype. The differentially expressed genes are enriched in pathways that are directly or indirectly related to β‐thalassemia such as hemopoiesis, heme biosynthesis, response to oxidative stress, inflammatory responses, immune responses, control of circadian rhythm, apoptosis, and other cellular activities.

**Conclusion:**

We compare our findings with published results of RNA‐sequencing analysis of sickle cell disease and erythroblasts from a KLF1‐null neonate with hydrops fetalis, and recognize similarities and differences in their transcriptional expression patterns.

## 1 INTRODUCTION

The hemoglobinopathies are endemic in many world populations, and have genetic carrier rates exceeding 40% in Africa and Southeast Asia (Modell & Darlison, 2008). These disorders include mutations in globin coding sequences that lead to structural changes in encoded proteins, such as the sickle hemoglobin, and mutations that alter the expression of the α‐ and β‐globin genes such as the thalassemias. Although the etiology of β‐thalassemia is well understood, the disease is highly heterogeneous at both the molecular and clinical levels. Approximately 200 β‐thalassemia alleles have been characterized and archived in the Var database (Patrinos et al., 2004). Unlike α‐thalassemia, in which the vast majority of mutations are large deletions in the α‐globin gene cluster, β‐thalassemia is usually caused by mutations involving one or a few nucleotides in β‐globin gene (HBB) or its immediate flanking regions. Mutations that completely abolish expression of the HBB are designated β^0 ^alleles, while other mutations in HBB cause varying degrees of quantitative reduction in β‐globin expression and are classified as β^+^ or β^++^ alleles (Thein, 2005). An imbalance between α and β chains causes accumulation of aberrant β_4_ tetramers in α‐thalassemia, or the toxic aggregation of α‐globin in β‐thalassemia (Khandros, Thom, D'Souza, & Weiss, 2012), both result in anemia due to hemolysis compounded by a cellular stress‐response and ineffective erythropoiesis (Ribeil et al., 2013). Hemolysis leads to physiological iron overload, particularly in thalassemia major. The elevated stores of iron may be a key factor in increased inflammation and susceptibility to infection (Wanachiwanawin, 2000). Infection is among the most common causes of death in individuals with β‐thalassemia (Miller, Dykes, & Polesky, 1988).

Currently, individuals with β‐thalassemia are assessed clinically by measurement of blood indices, electrophoresis of hemoglobin (Hb) molecules in peripheral blood, and physical examination. A better understanding of the disease could develop through the study of patterns of transcription in blood cells of individuals with β‐thalassemia, as these findings could indicate the major areas of compensatory or aberrant regulation that accompany the primary DNA sequence defects. Here, we studied transcriptome profiles of two members of a family with β‐thalassemia carrying a novel mutation in the HBB. This is the first use of RNA sequencing to investigate broadly the blood cell gene expression in β‐thalassemia in humans. We compare our findings with published results of RNA‐sequencing analysis of sickle cell disease (SCD) and erythroblasts from a KLF1‐null neonate with hydrops fetalis, and recognize similarities and differences in their transcriptional expression patterns.

## 2 MATERIALS AND METHODS

### 2.1 Editorial policies and ethical considerations

The collection of samples for this study was approved by the Pasteur Institute of Iran, application number 6266. Consent to participate in the study was contained in the written form signed by family members or guardians. A written informed consent for publication of personal information, including clinical descriptions and test results was obtained for each family member at the Pasteur Institute of Iran.

### 2.2 Case description and clinical examination

The subjects were a 5‐year‐old girl and her parents. Both parents were clinically asymptomatic for β‐thalassemia, but clinical examination of their daughter showed anemia, paleness, jaundice, and splenomegaly. She also had a history of transfusion dependence but her most recent transfusions were in the years before this study was undertaken, so the transfused cells are not expected to interfere with the present study. A complete blood count and Hb analysis were carried out for the family according to standard methods. Molecular studies were undertaken on both genomic DNA and RNA isolated from family members, after obtaining informed written consent. The mother's samples were labeled “M”, the daughter's samples were labeled “D”, and control samples were labeled “N”.

### 2.3 Mutation detection in the β‐globin gene

Following the isolation of genomic DNA using a standard salting‐out procedure (Miller et al., 1988), the entire HBB gene was polymerase chain reaction (PCR)‐amplified and the nucleotide sequence was determined, using two primer sets encompassing exons 1 and 2 (fragment A: Beta1F 5'‐ GGG CCA AGA GAT ATA TCT TAG‐3', Beta1R 5'‐AATGACATGAACTTAACCATAG‐3') and another set encompassing exon 3 and the 3'UTR (fragment B: Beta2F 5'‐GCA CCA TTC TAA AGA ATA ACA G‐3', Beta2R 5'‐GTT TGA ACT AGC TCT TCA TTT C‐3'). The PCR product was purified using a QIAquick PCR Purification Kit (Qiagen), and it was subjected to sequencing by the chain termination method on an ABI 3730 XL sequencer (Applied Biosystems, Foster City, CA, USA). The nucleotide numbering is based on GenBank accession number U01317. Investigations of the hemoglobin A (HBA) genes were carried out by both DNA sequencing and by screening for common α‐globin gene deletions (‐α^3.7^, ‐α^4.2^, ‐α^20.5^, and ^MED^) using a standard Gap‐PCR method (Chong, Boehm, Higgs, & Cutting, 2000).

### 2.4 RNA isolation and RT‐qPCR

Total RNA was isolated from blood using EDTA as an anticoagulant and Trizol reagent (Sigma‐Aldrich, Germany) following the manufacturer's instructions. One microgram of total RNA was used for cDNA synthesis with a QuantiTect reverse transcription kit (Qiagen). The concentration and quality of the cDNA from each sample were determined by absorbance at 260 and 280 nm, using a Nanodrop 2000c spectrophotometer (Thermo Fisher Scientific, Wilmington, DE). Relative quantification of β‐globin cDNA was performed by real‐time quantitative polymerase chain reaction (qPCR) using an iTaq^™^ Universal SYBR Green Supermix Kit (Bio‐Rad) following the manufacturer's instructions. Briefly, qPCR was performed in a 10‐µl volume, containing 5 µl SYBR Green Supermix, 2 µl cDNA (40 ng), 1 µl nuclease‐free water, and 1 µl each of forward and reverse primers (3 µM). Primer pairs for β‐globin cDNA (forward 5’‐GAT GAA GTT GGT GGT GAG GCC‐3’ and reverse 5’‐GCC CAT AAC AGC ATC AGG AGT G‐3’) as the target gene, and both ACTB (forward 5’‐TGG CAC CAC ACC TTC TAC AAT G‐3’ and reverse 5’‐GGT CTC AAA CAT GAT CTG GGT CA‐3’) and GAPDH (forward 5’‐GGA AGG TGA AGG TCG GAG TC‐3’ and reverse 5’‐ACA TGT AAA CCA TGT AGT TGA GGT‐3’) as the endogenous reference genes were designed to obtain amplified sizes between 70 and 150 bp for the maximum qPCR efficiency, as recommended by Bio‐rad.

The qPCR was performed on a CFX96 Touch^™^ Real‐Time PCR Detection System (Bio‐Rad) following a thermal cycling profile of 95°C for 3 min, and 39 cycles of 95°C for 10 s and 57°C for 30 s. Samples were analyzed in triplicate, and the expression of HBB relative to reference genes was normalized to a control sample from a healthy individual with normal hematological parameters and hemoglobin profile. The relative fold change was calculated using the method of Pfaffl (Robinson et al., 2011). Bio‐Rad CFX Manager 3.0 software was used for amplification and melting curve analyses.

### 2.5 Library construction and RNA sequencing

A quantity of 500 ng of the total RNA from each sample was used for RNA‐sequencing library preparation, and the quality of RNA was determined with a Bioanalyzer 2100. Libraries for RNA sequencing were prepared with a KAPA Stranded RNA‐sequencing Kit (Illumina), following the manufacture's instructions. Sequencing was performed on an Illumina HiSeq 2500 system for pair‐ended reads with a length of 100 bp (2 × 100 bp). Preprocessing and data quality control were performed using Illumina proprietary software.

More than 39 million 100 bp pair‐ended reads were generated for each sample. Reads were aligned to the human reference genome with the *STAR* genome index (Quinlan & Hall, 2010), which included both the genome sequence (GRCh37.71) and the exon/intron structures of known reference sequence (RefSeq) transcripts. On average, 80% of the reads (using a similarity score of 66%) were aligned successfully with the genomic sequence.

*Samtools *(Trapnell et al., 2012) was used to sort and index the alignment files for visualization with the Integrative Genomics Viewer tool (Casero et al., 2015). The sorted binary sequence analysis file (BAM files) was also used to generate UCSC Browser tracks with a *genome Coverage Bed* from *Bed Tools *(Dennis et al., 2003). To this end, coverage files were normalized using the total signal for each sample.

The *cufflinks* (Senapathy, Shapiro, & Harris, 1990) suite of tools (version 2.2.1) was used for differential gene expression analysis, which provides and compares gene expression levels in units of Fragments Per Kilobase of transcript per Million mapped reads (FPKMs). The mapped reads were counted for RefSeq transcripts using *cuffquant*. The *cuffquant* outputs were passed to *cuffdiff* to test for significant differences in transcript abundance for each pair‐wise comparison between the three samples (N [Normal control], M [Mother], and D [Daughter]). Genes were flagged as differentially expressed when the log2|fold change| was >1 and the adjusted *p*‐value was <0.05. To allow cuffdiff analysis for a highly expressed gene, a default of a maximum of 1,000,000 fragments was used. This allows computation of the FPKM values for differentially expressed genes (DEGs), and allows comparison between expression levels of DEGs. The expression estimates were computed with HTSeq35 (unique hits and “intersection‐nonemtpy” mode for version v0.6.1p2) to generate gene‐level counts for each sample (Thein, 2013).

### 2.6 Analyzing effects of globin gene masking on gene expression analysis by RNA sequencing

One difficulty in analyzing gene expression in whole blood is that the majority of transcripts (over 70%) are from globin genes. The results of RNA‐sequencing analysis from whole blood were masked using *cufflinks* (*‐M/–mask‐file<mask.(gtf/gff)>*) to prevent interference from all the globin genes: HBA1, HBA2, HBB, HBD, HBG1, HBG2, HBE1, HBM, HBQ, and HBZ.

### 2.7 Gene ontology (GO) analysis using the DAVID annotation database

The Database for Annotation, Visualization and Integrated Discovery (DAVID) was used to determine the molecular functions, ontology terms, and the pathways significantly enriched in the DEGs among our samples (Li et al., 2015).

## 3 RESULTS

### 3.1 Hematological indices and genomic DNA sequence analyses

**Figure 1:**
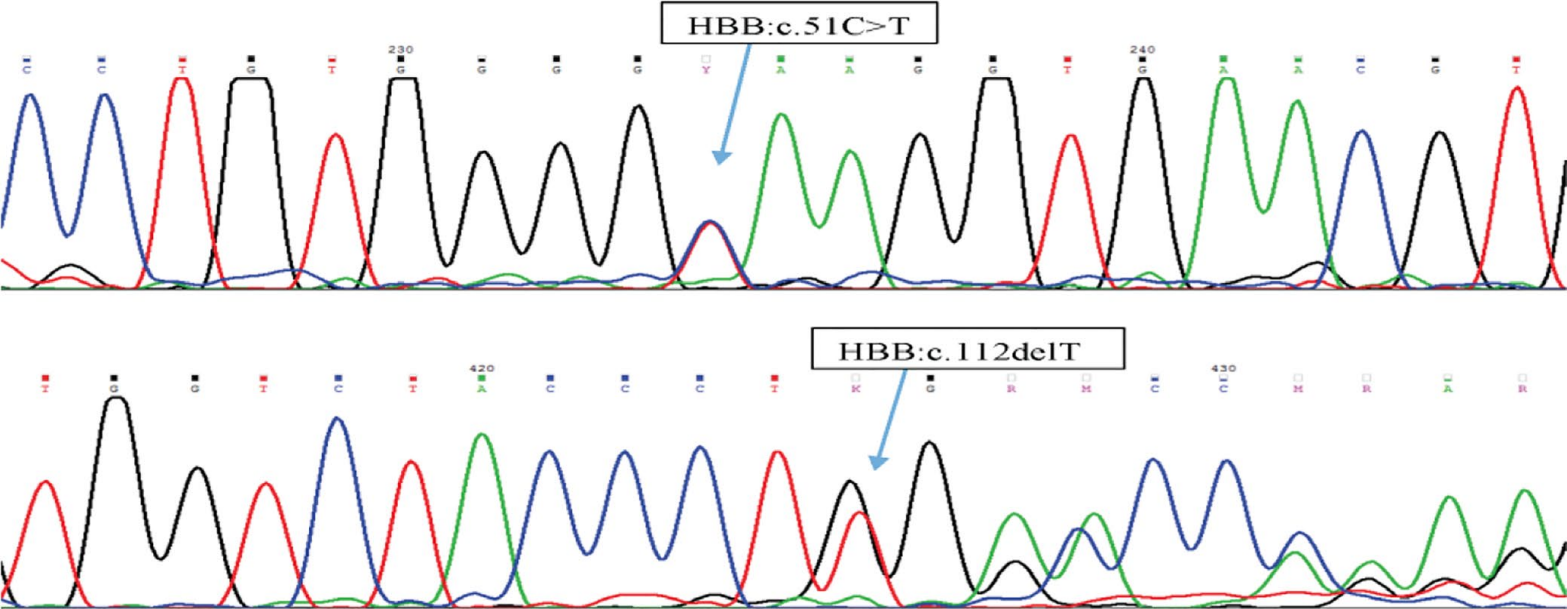
Sequencing of β‐globin genomic DNA from the daughter (D), with the top panel highlighting the novel maternally derived mutation (HBB:c.51C>T) and the bottom panel highlighting the paternally derived mutation (HBB:c.112delT)

Whole blood specimens were collected from a family in which the father (F) and daughter (D) were anemic, with hematological indices consistent with β‐thalassemia minor and β‐thalassemia intermedia, respectively. The mother (M) had a slight microcytic anemia with borderline indices (Table 1). Genomic DNA sequencing indicated that the father is heterozygous for a common β‐thalassemia allele in which a thymidine nucleotide is deleted from codon 37 of the β‐globin gene (HBB:c.112delT), resulting in a translational frameshift and early termination of translation (Figure 1). Sequencing of the mother's DNA revealed that she is a carrier of a novel mutation in exon 1 of HBB, in which there is a C to T transition mutation (HBB:c.51C>T) in the third nucleotide of codon 16. The daughter (D) is a compound heterozygote for the maternal and paternal mutations, and has the most severe phenotype.

**Table 1:** Hematological parameters of the family

Case and genotype	Age (years)	HGB (g/dl)	MCV (fl)	MCH (pg)	MCHC (g/dl)	RBC (106/μl)	HBA (%)	HBA2 (%)	HBF (%)
Mother (M)HBB:c.51C>T	28	12.8, 13.4	78.2	25.9	33.1	5.18	96.2	3.4	0.4
Father (F)HBB:c.112delT	35	10.5, 11.2	60.5	19.6	32.4	5.72	94.4	5	0.6
Daughter (D)HBB:c.51C>T HBB:c.112delT	5	7.1, 7.6	56.5	17.3	30.6	4.16	93.5	4.8	1.7

Abbreviations: HBA, hemoglobin A; HBA2, hemoglobin A2; HBF, fetal hemoglobin; HGB, hemoglobin; MCH, mean corpuscular hemoglobin; MCHC, mean corpuscular hemoglobin concentration; MCV, mean corpuscular volume; RBC, red blood cells.

### 3.2 Novel mutation introduces a cryptic splice donor site, and is associated with downregulation of the β‐globin gene

**Figure 2:**
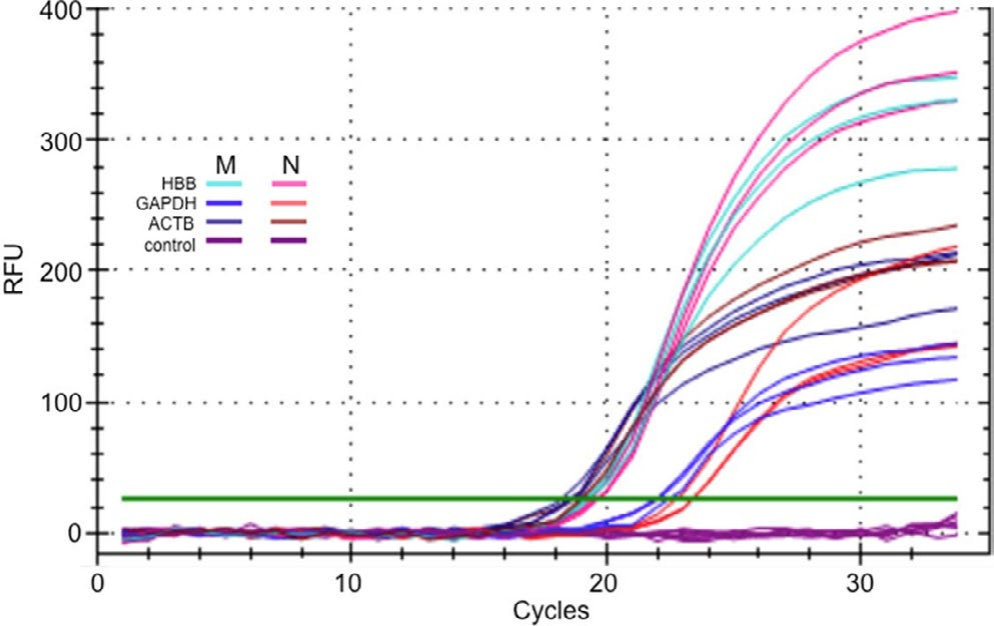
RT‐qPCR amplification curves for the measurement of the relative expression of HBB in the mother (M) as compared to normal sample (N). Additional controls include GAPDH and ACTB as known reference amplicons, and no added cDNA as negative controls. The horizontal green line indicates the threshold for comparative analysis between qPCR samples. The PCR cycle number is indicated on the *x* axis, and the relative fluorescence units (RFU) are indicated on the *y* axis. RT‐qPCR, real‐time quantitative polymerase chain reaction

Analyses of the mRNA sequence and splicing patterns of HBB were conducted in our laboratory by RNA‐sequencing analyses and RT/qPCR. The relative expression of β‐globin in M, as estimated by the cDNA counts during RNA sequencing, was approximately 70% of that detected in N (the ratio of normalized sequence reads was 285,022/410,599). The downregulation of β‐globin in M was also confirmed by RT/qPCR analyses, showing that the relative normalized expression of β‐globin in M was 0.77 (±0.1) of that detected in N (Figure 2). This slight downregulation of the β‐globin gene is consistent with the functional effect of the previously known cryptic splice site mutations at the HBB (Allan, Beattie, & Flint, 2008).

**Figure 3:**
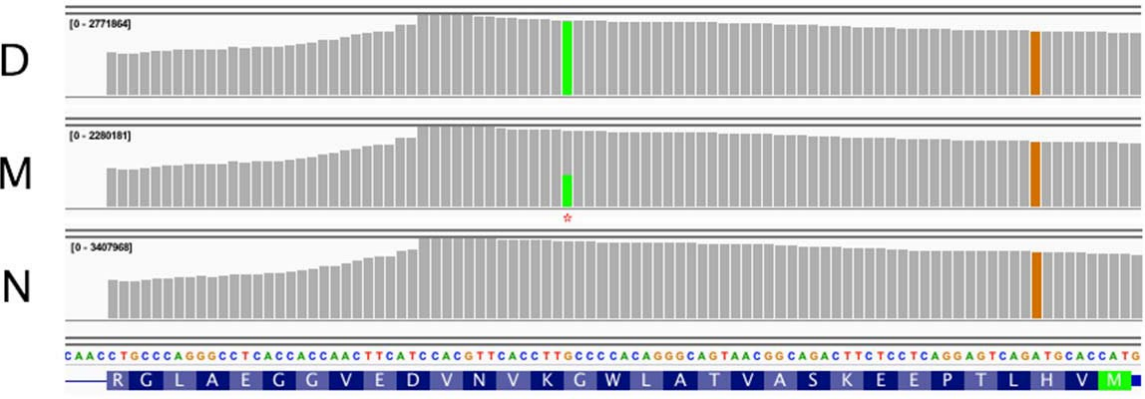
Steady‐state β‐globin transcript levels in the daughter (D), mother (M) and normal control (N). The reference sequence shown at the bottom is antisense β‐globin, with the amino acid translation written right‐to‐left, starting from the initiator methionine codon (green). The top three panels are visualizations of the exon 1 genomic sequencing coverage for the three samples, D, M, and N, with the height of the bar chart indicating the depth of the reads at each position. The novel mutation in codon 16 (GGC → GGT) is seen in the coverage tracks of both sample D and sample M (the green bar, marked with an asterisk), while a common SNP in codon 3 is present in all samples (orange)

While the allele expression difference was minor in M and consistent with the mother's slight microcytic anemia, the C>T transition represented 99% of the HBB transcripts in the compound heterozygote D (Figure 3). The strong prevalence of RNA transcribed from the maternal allele (HBB:c.51C>T) in the daughter was likely caused by preferential decay of the mRNA transcribed from the paternally derived allele (HBB:c.112delT) carrying a frame‐shift mutation in codon 37.

**Figure 4:**
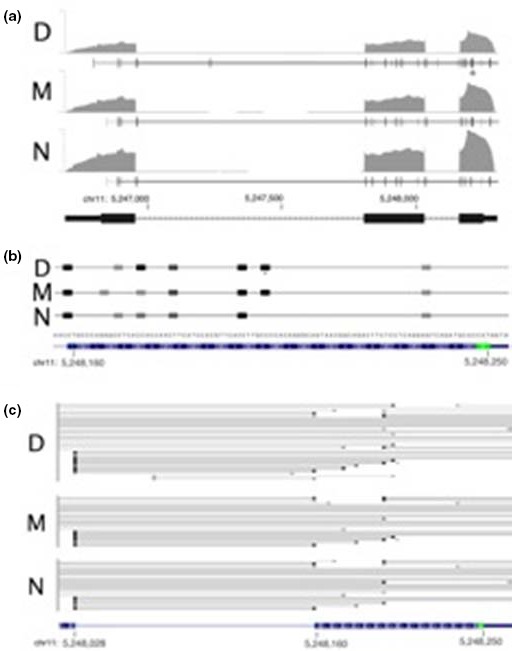
Gene expression at the HBB locus. (a) Normalized, whole‐genome RNA‐Seq coverage tracks are shown for D, M, and N. An asterisk represents the position of the novel mutation. Vertical black lines represent sequence reads of high abundance, and grey vertical lines represent reads of low abundance, showing their splicing locations. (b) Splice site usage in the exon 1 region of HBB, from samples D, M, and N. (c) A detailed view of the cDNA sequence reads spanning exon 1 (right) and exon 2 (left) of HBB, in samples D, M, and N. The horizontal lines indicate the sequence joints generated by splicing of these exons, and the canonical exon 1–exon 2 junction is shown at the bottom of the “N” examples. The cryptic splice donor site used in samples D and M are indicated by red asterisks. Dark and light bars indicate more or less frequently detected sequence reads, and reference information for this exon 1, intron 1, exon 2 region are indicated at the bottom

RNA‐sequencing reads with at least one gap in their genomic alignment were analyzed to establish the (putative) splicing patterns at the HBB locus. In both M and D, transcripts were detected that carried the HBB:c.51C>T mutation and spanned the novel joint of the putative splice site, confirming its use as a splice donor (Figure 4a–c). These aberrantly spliced products were found at a low frequency compared to the canonically spliced reads, but without information on the degradation of alternatively spliced mRNAs it is difficult to draw a conclusion about the exact extent of use of the cryptic splice site. It is noteworthy that a natural cryptic splice site spanning codon 17–19 is frequently used for splicing in all three samples (Figure 4b,c).

### 3.3 More than 300 genes are differentially expressed in β‐thalassemic blood

RNA‐sequencing analysis was used to characterize the expression of alpha and other β‐like globin genes in D and M, to develop an understanding of compensatory gene expression in these individuals. Compared to a normal individual, the HBD gene encoding δ‐globin was upregulated approximately 80‐fold in D, and the HBG1 and HBG2 genes encoding γ‐globin were upregulated 18‐ and 13‐fold, respectively. It is possible that HbA2 and HBF were generated in substantially increased amounts to compensate for the defective production of HbA in D. The expression of HBA1 and HBA2 encoding α‐globin chains was represented at slightly higher FPKM levels in D, compared to N, perhaps as a consequence of heightened erythropoiesis rather than gene regulation at the cellular level.

**Figure 5:**
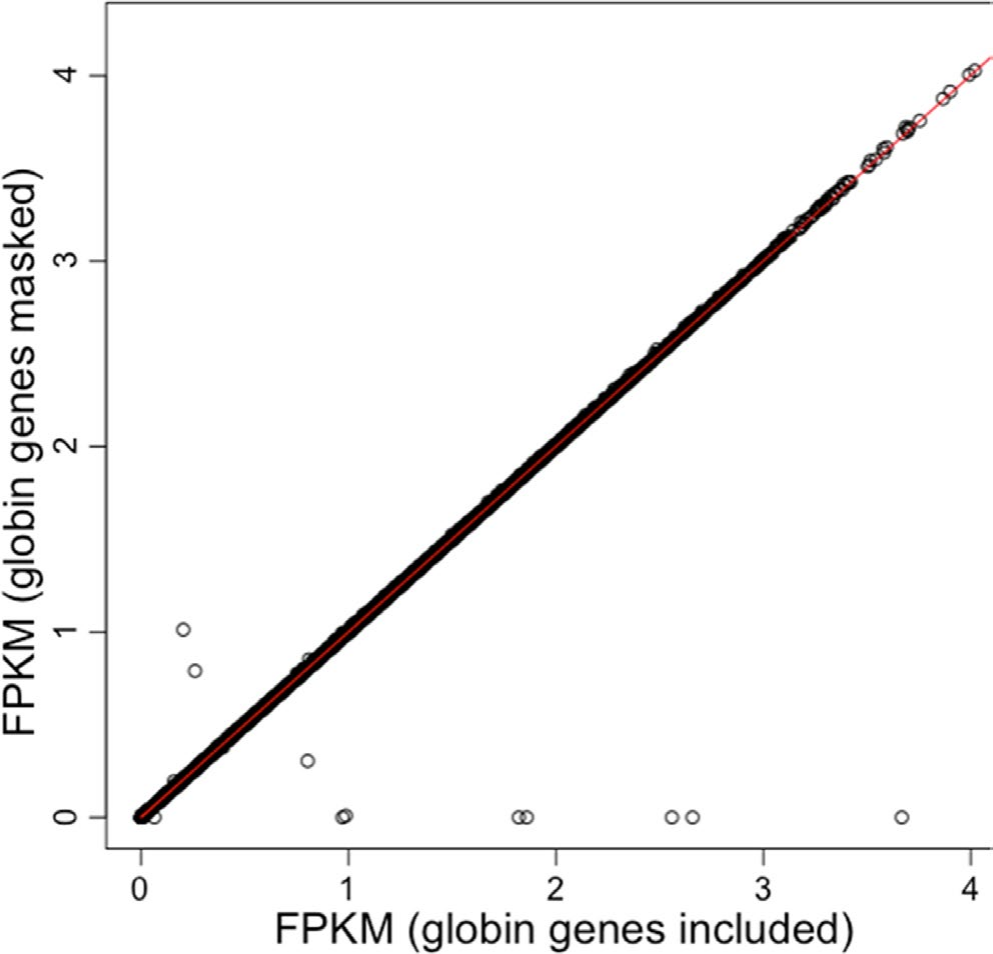
Including highly expressed globin genes in a broad transcript analysis does not typically change the FPKM values using the *cuffdiff* software. Comparison of the log FPKMs of all sequenced genes in a normal sample N, with (vertical axis) and without (horizontal axis) masking of human globin genes. The eight off‐diagonal genes along the bottom are all globin family genes, whereas the two above the diagonal are the genes SMN2 and EIF3CL. The red line indicates the broad linear correlation (*R* > 0.997). FPKMs, fragments per kilobase of transcript per million mapped reads

The DEGs in samples M and D, compared to N, were determined using the *cuffdiff* analysis tool with the software settings adjusted to include highly expressed genes, as described in Materials and Methods. A separate analysis was performed to determine whether masking globin genes as targets in the assignment of reads using the *cuffdiff* software changes the FPKM value for non‐globin genes. There was a high correlation of FPKM values (*R* > 0.997) between the two approaches (masking vs. not masking) for all but 10 genes, as shown in Figure 5. The genes below the diagonal are globin genes that appear to have null expression when their genomic targets are masked. The two genes above the diagonal are SMN2 and EIF3CL, which show an artificial increase in apparent expression when globin gene targets are masked. These may have adventitious similarity to globin reads and serve as an alternate target for *cuffdiff*. As the remaining genes lie closely along the diagonal, the masking (or not) of globin genes in the analysis does not appear to significantly bias the FPKM counts for non‐globin genes.

**Figure 6:**
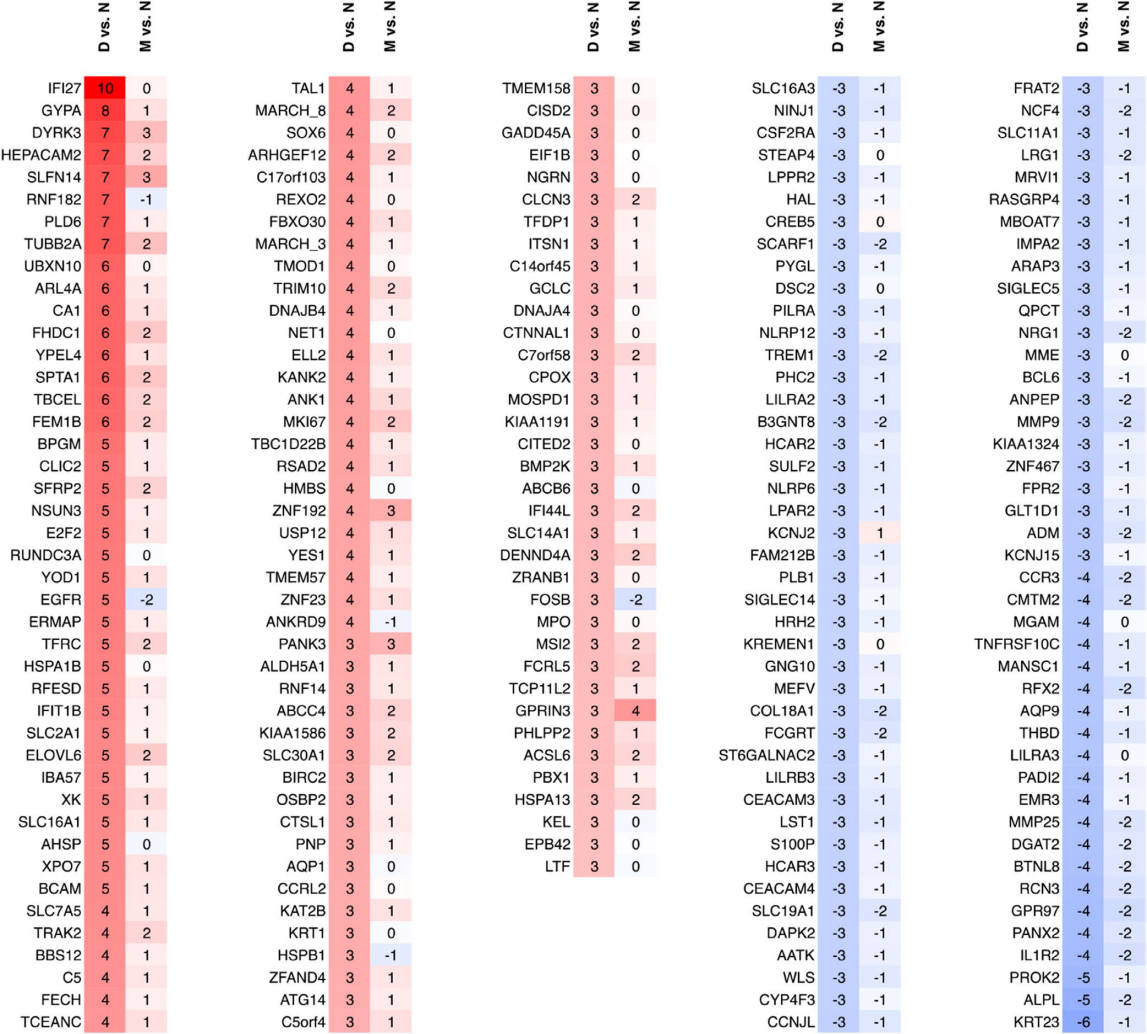
Significantly differentially expressed genes demonstrated by pairwise comparisons of samples N, M, and D, using the software package *cuffdiff*. The heat‐map is divided into five parts, and in each part, the first and second columns represent the data for, respectively, D versus N and M versus N pairwise comparisons. A red color indicates upregulation, and a blue color indicates downregulation. The numbers in the cells show the log base 2 of the fold change (numbers are rounded). The heat map is generated using selected genes with differential expression greater than ~6× (log2 >2.5) or less than ~1/6 (log2 < −2.5). Also, the genes with log2 (fold change) equal to inf or –inf have been omitted. The red and blue colors indicate upregulation and downregulation respectively.

In pairwise comparisons between the samples M, D, and N, there were a total of 398 significant DEGs, 312 of which were differentially expressed in sample D compared to N. There were only 37 DEG by comparison of sample M with N, and 177 that were differentially expressed between D and M, in accordance with the severe hematological changes seen in the daughter compared to the slight anemia present in the mother. Of the 398 DEG in the overall list, 343 had expression levels with FPKM > 2 in all three comparisons (Table 2). The differential patterns of gene expression in samples D and M are shown in Figure 6, and depend on both changes in the cellular make‐up of the blood and changes in gene expression within individual cell types. An increase in erythropoiesis, for example, in the daughter who is severely anemic, would result in an increase in erythroblast‐associated transcripts in the total pool of buffy‐coat RNA. The overall RNA‐sequencing pattern is thus a snapshot of the heterogeneous blood compartment as a whole, and indicates the physiological state of that tissue.

**Table 2:** Selected gene ontology terms that were significantly enriched among the differentially expressed genes in the daughter (D)

Ontology terms	Genes	*p*‐value	Gene examples
Erythrocyte homeostasis	8	3.2 × 10^−5^	BPGM, BCL6, KLF1, SOX6, TAL1, DYRK3, EPB42, TRIM10
Hemopoiesis; immune system development	15	1.4 × 10^−4^	BPGM, BCL3, BCL6, KLF1, RASGRP4, SOX6, TAL1, DYRK3, EGR1, EPB42, AHSP, MMP9, PBX1, SPTA1, TRIM10
Iron ion binding	18	8.1 × 10^−5^	CISD2, RFESD, STEAP4, C5orf4, CYP1B1, CYP4F3, FECH, HBA2, HBA1, HBB, HBE1, HBG1, HBM, HBQ1, ISCA1, LTF, MPO, RSAD2, SLC11A1
Hemoglobin chaperone	6	6.0 × 10^−6^	GATA1, CPOX, AHSP, FECH, HBB, HMBS
Inflammatory response	14	7.8 × 10^−3^	CCR3, C5, FPR2, IL8, KRT1, LY96, MEFV, MMP25, ROK2, SLC11A1, TLR6, TLR8, TFRC, VNN1
Defense response	23	2.6 × 10^−3^	BCL3, MEFV, BPI, CCR3, C5, DEFA3, FPR2, IL8, KRT1, LTF, LILRA2, LILRA3, LILRB3, LY96, MMP25, MPO, PROK2, RSAD2, SLC11A1, TLR6, TLR8, TFRC, VNN1
Response to oxidative stress	8	3.4 × 10^−2^	CRYAB, EGFR, GCLC, JUN, KRT1, MPO, SELK, VNN1
Blood group antigen	8	6.0 × 10^−7^	ART4, KEL, XK, AQP1, BCAM, ERMAP, GYPA, SLC14A1
Response to bacterium	11	3.3 × 10^−3^	BCL3, ADM, BP1, DEFA3, IRAK3, JUN, LTF, LY96, SLC11A1, THBD, TLR6
Regulation of apoptosis	23	4.6 × 10^−2^	BCL3, BCL6, CITED2, NLRP12, ARHGEF12, BIRC2, COL18A1, CRYAB, DAPK2, DYNLL1, EGFR, FEM1B, GCLC, HSPB1, HSPA1B, HSPA1A, ITSN1, JUN, MMP9, MPO, NRG1, NET1, PROK2, VNN1
Gas transport	8	1.9 × 10^−8^	AQP1, CA2, HBA1, HBA2, HBB, HBE1, HBG1, HBM, BQ1
Iron ion homeostasis	5	4.3 × 10^−3^	ABCB6, EPB42, LTF, SLC11A1, TFRC
Chemotaxis (chemokine activity)	10	3.1 × 10^−3^	CMTM2, CCR3, CCRL2, CXCL5, CKLF, C5, FPR2, IL8, PLAUR, PROK2
Regulation of cell shape	5	1.8 × 10^−2^	ARAP3, CDC42EP2, EPB42, LST1, SPTA1
Immunoglobulin‐like fold	24	2.9 × 10^−4^	FCGRT, FCRL3, FCRL5, HEPACAM2, BCAM, BTNL8, CEACAM3, CEACAM4, ERMAP, EPB42, IL1R2, LRFN1. LILRA2, LILRA3, HLA‐DQA2, NRG1, NFATC2, OSCAR, PILRA, SIGLEC14, SIGLEC5, SIRPB1, SIRPB2, TREM1
Protein kinase cascade	13	4.5 × 10^−2^	BCL3, NLRP12, RASGRP3, C5, CRYAB, DAPK2, EGFR, LY96, LPAR2, MAP4K5, PROK2, TLR6, TLR8
Disulfide bond	70	1.5 × 10^−3^	KIAA1324, CTSL1, CCRL2, CXCL5, CLIC2, ST3GAL4, ST6GALNAC2, ALDH5A1, DLL3, DPEP2, EMR3, EMR3, IFI30, EMR3, HRH2, KREMEN1, LRG1, MGAM, MME, NTNG2, PLAUR, PTN, PRRG4, PTGDS, PTP4A3, SCARF1, SOSTDC1, SFRP2, TNFRSF10C
Carboxylic acid transport	8	2.0 × 10^−2^	XK, AQP9, CPT1B, SLC11A1, SLC16A1, SLC16A3, SLC19A1, SLC7A5
Membrane fraction	24	2.9 × 10^−2^	ABCC4, RASGRP4, ACSL6, NCEH1, CEACAM4, CPT1B, CSPG5, CSPG5, CYP4F3, DGAT2, DYNLL1, GYPA, HSPA13, ITSN1, KREMEN1, LNPEP, MME, SLC16A1, SLC16A3, SLC19A1, SLC2A1, STX3, YES1

### 3.4 Involvement of many DEGs in pathways relevant to the clinical management of β‐thalassemia

The DAVID Bioinformatics Resources tools were used to organize the list of DEGs under ontologic terms, so that genes with common functions could be clustered. As shown in Table 2, there were highly significant clusterings of genes involved in erythrocyte development and function. The dysregulation of globin genes, the cause of β‐thalassemia, is included under the ontologic term Iron Ion Binding. There are also multiple categories of the DEGs that are involved in hematopoiesis, response to oxidative stress, inflammation, immune response, protein modification, and apoptosis (Table 2). Although some DEGs belong to more than one ontologic category, almost all of the genes in erythrocyte homeostasis, hemopoiesis, iron ion binding, hemoglobin's chaperone, blood group antigens, and response to oxidative stress categories are upregulated. Conversely, most of the DEGs belonging to the defense response, response to bacteria, chemotaxis, and immunoglobulin‐like fold categories are downregulated.

The most significantly upregulated gene in D (>1,100‐fold, compared to N) was IFI27, an INFa inducible gene that encodes an intracellular product used as a marker of epithelial‐mesenchymal transition in many forms of cancer (Yu et al., 2016). Its upregulation in β‐thalassemia may be indicative of myelofibrosis, or an inflammation and scarring of the bone marrow, seen also in Philadelphia‐negative chronic myeloproliferative neoplasms where there is an exceedingly high level of expression of IFI27 in whole blood (Gorden et al., 2013). IGFBP5 (insulin‐like growth factor‐binding protein 5) is also one of the substantially upregulated genes in D (82‐fold, compared to N), and it has been observed to be upregulated in the fibrotic disorders systemic sclerosis/scleroderma and idiopathic pulmonary fibrosis. The product of the IGFBP5 gene is secreted and may play a causal role in epithelial cell senescence and fibroblast overgrowth (Cai et al., 2015). The IGFBP5 gene is expressed at low levels in cell lines of hematopoietic lineages, and the dramatic increase in its RNA levels in the blood compartment of D may reflect a derangement of the bone marrow or increased population of fibrocytes associated with inflammation elsewhere in the body (Mukaiyama et al., 2015).

The microenvironment of the bone marrow may be profoundly affected by the disease β‐thalassemia and some of the changes may be mediated by hyaluronic acid (HA), a major component of the extracellular matrix in bone marrow. For example, the receptor EGFR (epidermal growth factor receptor) is 35‐fold upregulated in D and is involved in fibroblast to myofibroblast differentiation, mediated by HA and in response to TGF‐β1 signaling (Lee et al., 2009). The extracellular matrix proteoglycans BCAN (brevican, 66‐fold upregulated in D) and NCAN (neurocan core protein, 47‐fold upregulated in D) have roles in fibrosis, inflammation, and wound recovery, and bind to HA (Gileles‐Hillel, Kheirandish‐Gozal, & Gozal, 2015). The upregulation of RAP1GAP (Rap1 GTPase‐activating protein) by 140‐fold in D may help reduce inflammation mediated by Rap1 and NFκB (Xi, He, Zhang, Xue, & Zhou, 2014), by returning Rap1 to an inactive (GDP bound) state.

Osteoporosis is one of the leading causes of morbidity in individuals with β‐thalassemia, and the ALPL (alkaline phosphatase) gene required for proper osteoblast function was 33‐fold downregulated in D compared to N. This reduction in expression may be a regulatory consequence of iron overload (Kramer et al., 2001). Dual specificity tyrosine‐phosphorylation‐regulated kinase 3 (DYRK3, 140‐fold upregulated in D) may also be important in bone homeostasis as it acts as a negative regulator of osteoclastogenesis in mice (Raghavachari et al., 2012). Solute carrier family 11A1 (SLC11A1, encoding a divalent metal ion transporter), was eightfold downregulated in D compared to N, which may also be a response to iron overload from hemolysis (Biggs et al., 2001).

We observed differential expression of genes involved in the regulation of circadian rhythms, for example the circadian sleep–wake cycle process. Sleep disturbances, and particularly sleep‐disorders of breathing have been frequently observed in hemoglobinopathies such as SCD and β‐thalassemia, and also in both iron overload and iron deficiency anemia (Kihm et al., 2002). In particular, PROK2, encoding prokineticin 2 protein, is 24‐fold downregulated in D compared to N, is expressed in the suprachiasmatic nucleus and may act as an output component to transmit behavioral circadian rhythm and regulate other circadian rhythms such as sleep–wake cycles, feeding, endocrine rhythms, and a suggestion of signaling circadian day (Gallagher, Liem, Wong, Weiss, & Bodine, 2005). In addition, EGFR (epidermal growth factor receptor) indirectly controls inhibited locomotor activity and disrupted circadian sleep–wake cycles via the alteration of TGF‐α (Nirupam et al., 2012), and it is 35‐fold upregulated in D.

**Figure 7:**
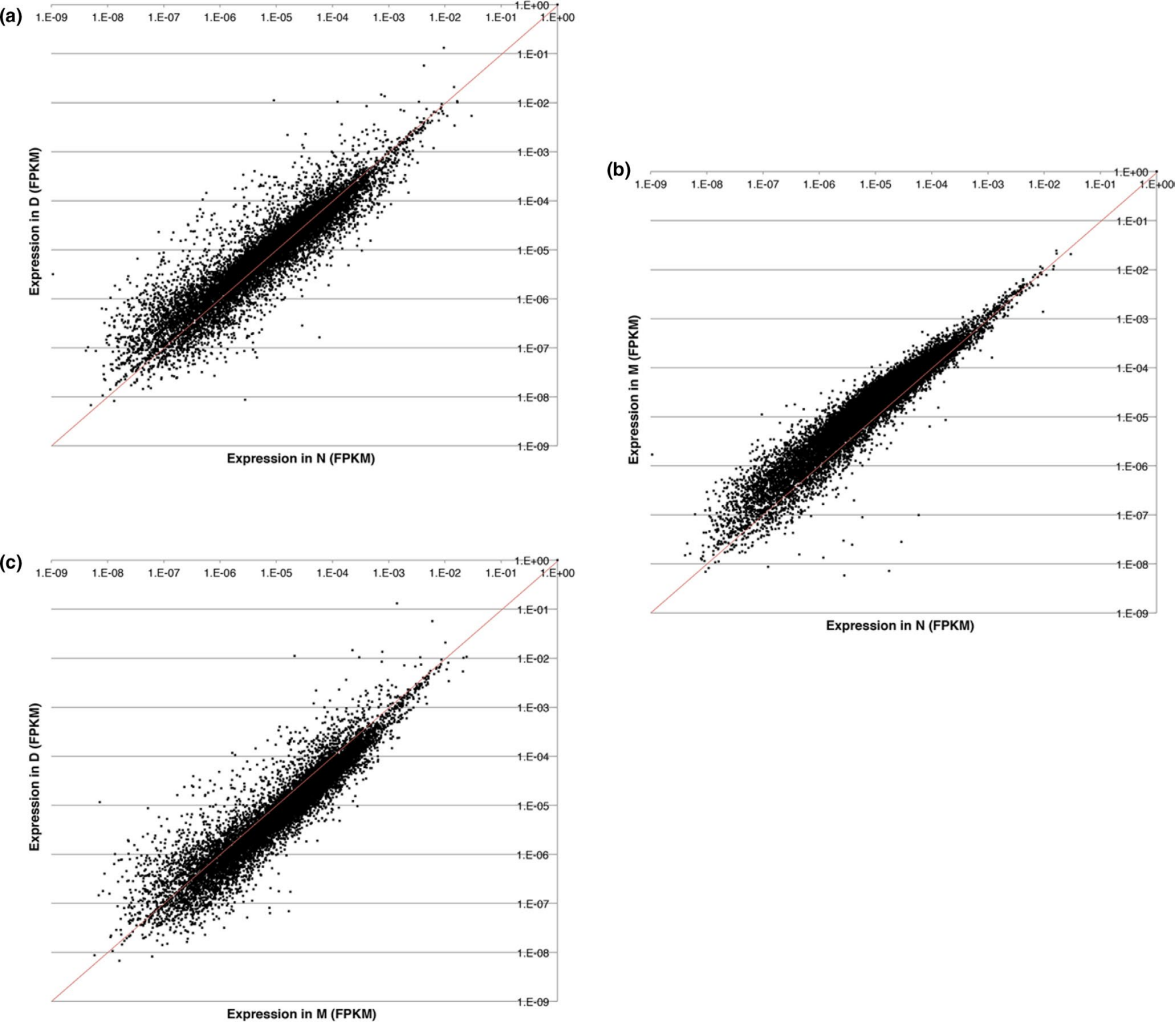
Diagonal (log–log) plots of expression levels of individual genes (FPKM units) among the three samples. (a) D versus N comparison, (b) M versus N comparison, and (c) D versus M comparison. The diagonal red lines indicate equivalence of expression. FPKMs, fragments per kilobase of transcript per million mapped reads

Scatter plots (log–log) were used to compare the relative expression of individual genes in samples D, M, and N over a wide range of expression (Figure 7a–c). An equivalence between FPKM values in the pairwise comparisons is shown as a diagonal line, and its offset from the data is likely to indicate a robust process of hematopoiesis in D and M compared to N. The comparison also indicates substantially greater numbers of genes that are up‐ or downregulated in D compared to M.

### 3.5 β‐Thalassemia shows important similarities and differences with SCD at the transcriptome level

The list of DEGs in β‐thalassemia was compared to the previously published RNA‐sequencing results for SCD (Raghavachari et al., 2012). In the case of SCD, there were 331 DEGs in whole blood from individuals with SCD compared to normal samples, and approximately one third of them (110 genes) were in common with the 343 DEG in β‐thalassemia, as shown in Table 3. Many genes involved in hematopoiesis, erythrocyte hemostasis, and iron‐binding proteins were differentially expressed in both β‐thalassemia and SCD, which might be expected since both disorders are hemolytic anemias. Improving the ability of erythrocytes to deliver oxygen to tissues would also be a predictable response in both β‐thalassemia and SCD, and upregulation of BPGM (bisphosphoglycerate mutase) is indeed seen in both cases. One of the genes upregulated in both β‐thalassemia and SCD was KLF1 (Kruppel‐like factor 1), a master regulator of red blood cell gene expression. Twenty‐five DEGs in both β‐thalassemia and SCD were also found in the published list of the DEGs in circulating erythroblasts from a homozygous KLF1‐null neonate with hydrops fetalis (Magor et al., 2015).

**Table 3:** Shared differentially expressed genes in the disorders β‐thalassemia, sickle cell disease, and KFL1‐null anemia

Disorders	Expressed genes
β‐Thalassemia and sickle‐cell anemia	ABCB6, ACSL6, ALPL, ANK1, ANKRD9, AQP9, RHGEF12, ARL4A, BCAM, BIRC2, BMP2K, BPGM, C14orf45, C5orf4, CA1, CA2, CCRL2, CISD2, CLCN3, CLEC4E, CLIC2, CTNNAL1, CYP4F3, DAPK2, DCUN1D1, DNAJB4, DOCK5, DYRK3, E2F2, ELL2, ELOVL6, EMR3, ERMAP, FAM83A, FECH, FHDC1, GCLC, GLT1D1, GPR97, GYPA, HAL, HBA1, HBA2, HBB, HBE1, HBG1, HBM, HEPA, CAM2, HMBS, HRH2, HSPA13, IFI27, IFI44L, IL1R2, ISCA1, ITSN1, KANK2, KCNJ15, KCNJ2, KEL, KIAA1324, KLF1, KRT1, KRT23, MANSC1, MAP4K5, MARCH3, MARCH8, MGAM, MME, MMP9, MOSPD1, NFIX, NSUN3, OSBP2, PLVAP, PPME1, REPS2, RFESD, RNF14, RNF182, RSAD2, RUNDC3A, SFRP2, SLC14A1, SLC16A1, SLC2A1, SLC45A4, SLC7A5, SOX6, SPTA1, STEAP4, TAL1, TBC1D22B, TBCEL, TCEANC, TCP11L2, TFDP1, TMEM14B, TMEM158, TNFRSF10C, TRAK2, TREM1, TRIM10, UBXN10, USP12, XK, XPO7, YPEL4, ZNF23
β‐Thalassemia and KLF1‐null anemia	ABCB6, ABCC4, AIDA, ANK1, ANKRD9, ARL4A, ART4, B3GNT8, BMP2K, C3orf58, C5, CLCN3, CLIC2, DCUN1D1, DYRK3, ERMAP, FBXO30, FHDC1, GYPA, HIST1H2BD
Sickle‐cell anemia and KFL1‐null anemia	ABCB6, ABCG2, ANK1, ANKRD9, ARL4A, BMP2K, C10orf10, C17orf99, CLCN3, CLIC2, DCUN1D1, DYRK3, ERMAP, FHDC1, FOXO3, GDF15, GYPA, GYPE, HBD, IGF2, IGF2BP2, KLC3, KRT1, LNX2, MOSPD1, NSUN3, RAP1GAP, RHD, RUNDC3A, SERPINI1, SLC14A1, SLC16A1, SLC6A19, SLC6A9, SLC7A5, TAL1, TBCEL, TCEANC, TCP11L2, TFR2, TSPAN7, USP12, XK, YIPF6
β‐Thalassemia, sickle‐cell anemia and KLF1‐null anemia	ABCB6, ANK1, ANKRD9, ARL4A, BMP2K, CLCN3, CLIC2, DCUN1D1, DYRK3, ERMAP, FHDC1, GYPA, KRT1, MOSPD1, NSUN3, RUNDC3A, SLC14A1, SLC16A1, SLC7A5, TAL1, TBCEL, TCEANC, TCP11L2, USP12, XK

The genes differentially expressed in β‐thalassemia but not in SCD included AHSP (alpha hemoglobin‐stabilizing protein), and the transcription factor GATA‐1 that induces AHSP expression (Gallagher et al., 2005; Kihm et al., 2002). AHSP prevents aggregation of α‐globin during erythroid cell development and is predicted to moderate pathological states of α‐globin excess such as β‐thalassemia. SCD is not associated with α/β chain imbalance, and so it is understandable that upregulated expression of AHSP is unnecessary. A surprising finding is that most genes differentially expressed in β‐thalassemia but not in SCD are involved in inflammation and immunity. Among these were genes that are activated in response to bacterial pathogens such as ADM, BCL3, BP1, DEFA3, IRAK3, JUN, LTF, LY96, SLC11A1, THBD, and TLR6 (see Data S1).

## 4 DISCUSSION

We described the downregulation of the β‐globin gene in β‐thalassemia by RNA‐sequencing analysis using a novel mutation in HBB in a family with an unexpectedly severe case of β‐thalassemia. The RNA‐sequencing analysis allows comparison of the effects of β‐thalassemia on β‐globin and other genes between β‐thalassemia and other hemoglobinopathies. We present results of sequencing analysis that show that the mother has a novel β‐globin mutation that introducing a cryptic splice cite that explains why her phenotype is very mild compared to the expected phenotype of a carrier parent for β‐thalassemia. The novel codon 16 mutation introduces a cryptic splice donor site in exon 1 and is associated with downregulation of β‐globin expression as confirmed by RNA‐sequencing analysis and qPCR. The newly described mutation contributes to a severe β‐thalassemia intermedia in a compound heterozygous daughter (D), but her mother (M) carrying the novel mutation in combination with the wild‐type allele is phenotypically asymptomatic and has borderline normal hematologic parameters. The moderate downregulation of HBB expression in M is consistent with the functional effect of the previously known cryptic splice site mutations at the HBB gene that exhibit a recessive pattern of inheritance (Allan et al., 2008). The expression of HBB in D is more complicated, since the paternal allele bears a frameshift mutation and the maternal allele introduces a cryptic splice site leading to a truncated protein product. While the steady‐state HBB transcript levels in D are approximately 93% of N, most arising from the maternal allele, a smaller subset of transcripts will encode a full‐length β‐globin protein.

Blood transcriptome analysis has not been previously reported for a human case of β‐thalassemia, and this study allows a better understanding of the regulatory changes and disease processes taking place in affected individuals. Over 300 genes were identified as being differentially expressed (DEGs) between the samples from D and M, in comparison with a normal sample (N). The DEGs in β‐thalassemia were interpreted through GO enrichment analysis. This revealed that the genes most affected in β‐thalassemia are involved in the hematopoietic response to oxidative stress, inflammation, immune response, protein modification, and apoptosis, as shown in Table 2.

A comparison of DEGs in β‐thalassemia versus SCD and KLF1‐null anemia shows that while many DEGs involved in response to hemolysis, iron homeostasis, and anemia were common to these disorders, over 200 DEGs were unique to β‐thalassemia. These results indicate fundamental differences between the anemias.

While this study only provides information on two individuals, a carrier mother and her severely affected daughter, it provides a wealth of data on how the disease is manifested in that family. For example, the expression of the gene IFI27 is upregulated more than 1,100‐fold in D and this may be indicative of a process of myelofibrosis or fibrotic scarring of the bone marrow. This has only been recently reported in a single case of β‐thalassemia (Nirupam et al., 2012), and it may be an overlooked element of the pathology of the disease. Exhaustion of the bone marrow epithelial microenvironment and subsequent fibroblast overgrowth would be predicted to interfere with the robust rate of erythropoiesis needed to replace cells lost to hemolysis, and therapeutic approaches that minimize or prevent myelofibrotic scarring might be helpful. Osteoporosis is a significant cause of morbidity in β‐thalassemia, and downregulation of ALPL expression by more than 30‐fold in D provides an important clue as to how that may develop. There are also indications that dysregulation of several regulatory genes (PROK2, EFGR) may be at the root of the sleep disturbances and apneas that are among the problems associated with β‐thalassemia. This study, the first genome‐wide RNA‐seq analysis of the blood transcriptome in β‐thalassemia, reveals some new molecular biomarkers responding to the disease, which can be examined in future studies as therapeutic targets to improve the quality of life for those affected with β‐thalassemia.

## CONFLICT OF INTEREST

The authors have no conflict of financial interest.

## AUTHOR'S CONTRIBUTION

FT carried out the molecular genetic studies, including qPCR, participated in the sequence alignment, and participated in drafting the manuscript. MH collected samples, conducted clinical studies, performed nucleotide sequence determination of the β‐globin genes, and multiplex ligation‐dependent probe amplification of the α–globin genes. GS conducted physical examination and initial clinical studies.
